# Supplementary material for: Genome-Wide Identification and Analysis on YUCCA Gene Family in Isatis indigotica Fort. and IiYUCCA6-1 Functional Exploration
Source: Int J Mol Sci. 2020 Mar 22;21(6):2188. doi: 10.3390/ijms21062188 (PMC7139497; doi:10.3390/ijms21062188)
Supplement: Supplementary file 1 [file ijms-21-02188-s001.pdf]

Supplementary File

Table S1 Primers sequences of all genes for qRT-PCR.

Primer Name	Forward Primer (5'-3')	Reverse Primer (5'-3')
IiYUCCA1	CCTCGTACACGGTCCCATAA	ATGGAGTTTGAGTCGGTCGT
IiYUCCA2	CCTTTCCCTTCAAGCTACCC	CGGGAAACATACTCCGTTGT
IiYUCCA3	AGACAGTCCAGTCAGCGAAA	ACTCAAACTCGCAAGAACCG
IiYUCCA4	AGAAGCAGGGCATAGTCGAA	GGAACGCCACGGTTTGATAA
IiYUCCA5-1	GACAAGGTCTTCTTCTTCCTCC	GCAAGAGTGGATCACCTCG
IiYUCCA6-1	CTGGAGGAGAGAGATGGAAGG	CTTTCAGACAAGCCGCCGT
IiYUCCA7	GACGTCGGAGCCTTCTCTAA	AAGTCGTTCTCCTTGAGCCA
IiYUCCA8	GAGCTCTAGCGAAGATCCGA	TGCGATAACCAGTAGCGAGT
IiYUCCA9	GATATTTATCAGACCGTCGGTGT	TTGTTGGGTATTCAGGGTAGTGA
IiYUCCA10	CGGGTTTAGCAACATCGGTT	TGGGCATAAAGGTGGGAACA
IiYUCCA11	CATTCCCTCCCAACACTCCT	ATACGCCGATTTGACGTTCC
IieIF2	TACCAGTGGCTCGCTTGAC	CAACCAAAGCAAATGACGTACTC
IiPP2A-4	GAATGCCTGCGAAAGTATGG	TCCTAATGTTGTCAAGGGTCTC
IiRPL15	GGGCGAAGAGGAAAGGTAG	CGGAAGACGGCGATAAAGAG
CaMV35S	ACAGAACTCGCCGTAAAGAC	GAAGGGTCTTGCGAAGGATA
NbActin	CGTTATGGTTGGAATGGGACAGAA	AAGAACAGGGTGCTCCTCGTGG
NbIAA8	ACCCTGCCAAAGATAGTGCT	GTGGTTGAACACGTGAGGTC
NbIAA16	TTGGCCACCAGTGAGATCAT	CTCCGTCCACGCTAACTTTC
NbGH3.1	TATGTACGCAGCTTCCGAGT	CACGGGATGAGTTAGGGTCA
NbGH3.6	CGTGTTTGCCTCAGGCTTTA	ACTGCCTCTCTCACTGAAGG
NbSAG12	CGTTATGGTTGGAATGGGACAGAA	AAGAA CAGGGTGCTCCTCGTGG

```
                10        20        30        40        50        60
1      ATGGATTTCTGCTGGAGGAGAGAGATGGAAGGTAAACTAGCACATGACTACTTGTCGTCC
1       M  D  F  C  W  R  R  E  M  E  G  K  L  A  H  D  Y  L  S  S

                70        80        90       100       110       120
61     GCAAAGAGCCATCACGGCGTGATGACGTCACCGCGCCGCGTCTGCGTCGTCACCGGACCG
21      A  K  S  H  H  G  V  M  T  S  P  R  R  V  C  V  V  T  G  P

               130       140       150       160       170       180
121    GTGATCGTAGGCGCCGGACCGTCCGGACTAGCGACGGCGGCTTGTCTGAAAGAGAAAGGA
41      V  I  V  G  A  G  P  S  G  L  A  T  A  A  C  L  K  E  K  G

               190       200       210       220       230       240
181    ATAACTTCGGTACTACTCGAGAGATCAAACTGCATAGCTTCACTATGGCAGCTTAAGACT
61      I  T  S  V  L  L  E  R  S  N  C  I  A  S  L  W  Q  L  K  T

               250       260       270       280       290       300
241    TACGACCGGCTCCATCTCCACCTTCCTAAGCAATTCTGTGAACTTCCGCTCATACCCTTT
81      Y  D  R  L  H  L  H  L  P  K  Q  F  C  E  L  P  L  I  P  F

               310       320       330       340       350       360
301    CCCGCCGATTTTCCAACTTATCCGACGAAGCAGCAGTTCATCGAGTACCTCGAGGACTAC
101     P  A  D  F  P  T  Y  P  T  K  Q  Q  F  I  E  Y  L  E  D  Y

               370       380       390       400       410       420
361    GCCCGGAGGTTCGATATACGGCCGGAGTTTGGTCAGACGGTTGAGTCGGCGGAGTTTGAT
121     A  R  R  F  D  I  R  P  E  F  G  Q  T  V  E  S  A  E  F  D

               430       440       450       460       470       480
421    GAGAACCTCGGGATGTGGCGCGTGACGAGCGTGGGTGAAGAGGGCACGACGGAGTACGTT
141     E  N  L  G  M  W  R  V  T  S  V  G  E  E  G  T  T  E  Y  V

               490       500       510       520       530       540
481    TGCCGGTGGTTGGTGGCTGCGACGGGGGAGAATGCGGAGCCGGTGGTCCCCAGGTTTGAG
161     C  R  W  L  V  A  A  T  G  E  N  A  E  P  V  V  P  R  F  E

               550       560       570       580       590       600
541    GGTATGGAGAAGTTTGAGGCCACCGGGATAGTTAAGCACACGAGTCATTATAAGACCGGC
181     G  M  E  K  F  E  A  T  G  I  V  K  H  T  S  H  Y  K  T  G

               610       620       630       640       650       660
601    GGAGATTTCGCCGGAAAAAGGGTTTTGGTCGTCGGATGTGGAAACTCCGGCATGGAGGTT
201     G  D  F  A  G  K  R  V  L  V  V  G  C  G  N  S  G  M  E  V

               670       680       690       700       710       720
661    TGTTTGGATCTCTGCAATTTCGGTGCTCAGCCTTCTCTCGTTGTCAGAGACGCTGTGCAC
221     C  L  D  L  C  N  F  G  A  Q  P  S  L  V  V  R  D  A  V  H

               730       740       750       760       770       780
721    GTCCTACCACGAGAGATGTTGGGTACTTCGACTTTTGGGCTGTCCATGTTGCTACTTAAA
241     V  L  P  R  E  M  L  G  T  S  T  F  G  L  S  M  L  L  L  K

               790       800       810       820       830       840
781    TGGTTGCCCATCCGGCTCGTTGACCGTTTCCTCTTGGTTGTTTCCCGGTTCATCCTCGGG
261     W  L  P  I  R  L  V  D  R  F  L  L  V  V  S  R  F  I  L  G

               850       860       870       880       890       900
841    GATACCACCCTGTTAGGTCTTAACCGTCCCCGTTTAGGCCCACTAGAGCTCAAAAATCTC
281     D  T  T  L  L  G  L  N  R  P  R  L  G  P  L  E  L  K  N  L

               910       920       930       940       950       960
901    ACCGGAAAAACGCCGGTTCTCGACGTCGGGACGCTTGCCAAGATCAAAACCGGAGATATC
301     T  G  K  T  P  V  L  D  V  G  T  L  A  K  I  K  T  G  D  I

               970       980       990      1000      1010      1020
961    AAGGTGTGTTCCGGGATAAGAAGGTTCAAACGGCATGAAGTTGAGTTCGATAACGGAAA
321     K  V  C  S  G  I  R  R  F  K  R  H  E  V  E  F  D  N  G  K

              1030      1040      1050      1060      1070      1080
1021   ACAGAGAGATTTGACGCCATAATATTGGCAACTGGCTACAAAAGCAACGTACCCTCTTGG
341     T  E  R  F  D  A  I  I  L  A  T  G  Y  K  S  N  V  P  S  W

              1090      1100      1110      1120      1130      1140
1081   CTAAAGGAGAATAAAATGTTTAGTAAGAAAGATGGATTCCCAATACAAGAGTTTCCGGAG
361     L  K  E  N  K  M  F  S  K  K  D  G  F  P  I  Q  E  F  P  E

              1150      1160      1170      1180      1190      1200
1141   GGATGGAGAGGGGAATGTGGGCTATATGCTGTCGGATTCACAAAACGTGGAATTTTCGGA
381     G  W  R  G  E  C  G  L  Y  A  V  G  F  T  K  R  G  I  F  G

              1210      1220      1230      1240      1250      1260
1201   GCATCAATGGATGCAAAGAAAATAGCTCAAGACATATACGAGTGCTCAAGAAAATCTGAT
401     A  S  M  D  A  K  K  I  A  Q  D  I  Y  E  C  S  R  K  S  D

              1270      1280      1290      1300      1310      1320
1261   CAAGCCCATAGACATATACAAGTGTTCATGTCAAGAAAACCTGATCAAGCCTATAGTAGA
421     Q  A  H  R  H  I  Q  V  F  M  S  R  K  P  D  Q  A  Y  S  R

              1330
1321   TTACTAGACGGTTGA
441     L  L  D  G  *
```

Figure S1. The nucleic acid and amino acid sequences of *IiYUCCA6*-1.

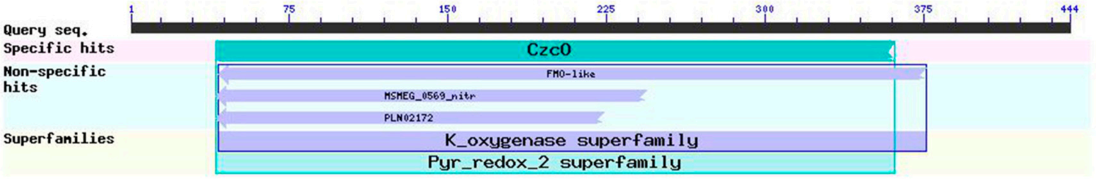

Figure S2. The conserved domain of IiYUCCA6-1.

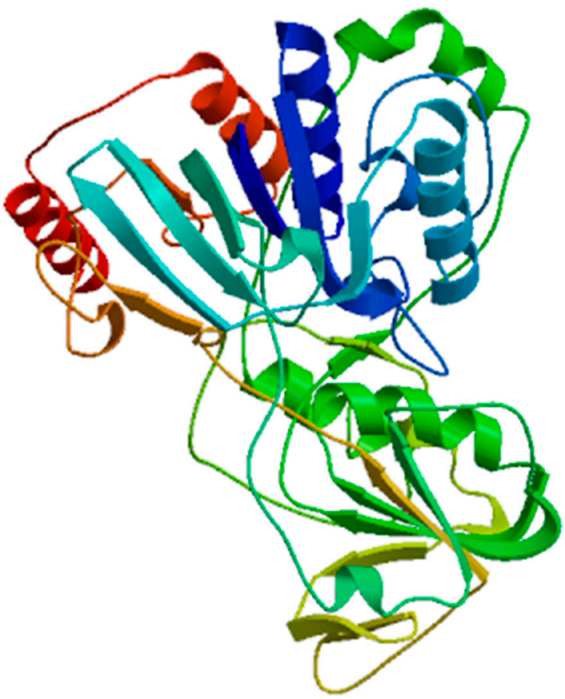

Figure S3. The predicted protein tertiary structure of IiYUCCA6.

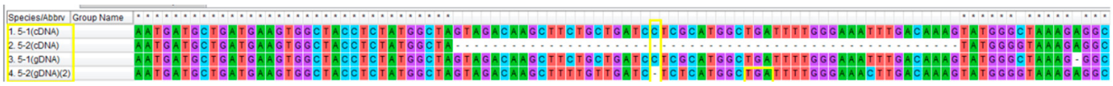

Figure S4. The sequence comparison of *IiYUCCA5-1* and *IiYUCCA5-2* based on *I. indigotica* genome data.
